# Clinical outcomes of WBRT plus EGFR-TKIs versus WBRT or TKIs alone for the treatment of cerebral metastatic NSCLC patients: a meta-analysis

**DOI:** 10.18632/oncotarget.19054

**Published:** 2017-07-06

**Authors:** Hong Zheng, Quan-Xing Liu, Bin Hou, Dong Zhou, Jing-Meng Li, Xiao Lu, Qiu-Ping Wu, Ji-Gang Dai

**Affiliations:** ^1^ Department of Thoracic Surgery, Xinqiao Hospital, Third Military Medical University, Chongqing 400037, China

**Keywords:** WBRT, EGFR-TKIs, NSCLC, BM

## Abstract

Whether WBRT plus EGFR-TKIs has a greater survival benefit than EGFR-TKIs alone or WBRT alone remains controversial in NSCLC patients with multiple brain metastases. To rectify this, we conducted a systematic meta-analysis based on 9 retrospective studies and 1 randomized controlled study published between 2012 and 2016, comprising 1041 patients. Five studies were included in the comparison of WBRT plus EGFR-TKIs and EGFR-TKIs alone. The combined HR for OS of patients with EGFR mutation was 1.25 [95% CI 0.98–2.15; *P* = 0.08] and for intracranial PFS was 1.30 [95% CI 1.03–1.65; *P* = 0.03], which revealed that EGFR-TKIs alone produced a superior intracranial PFS than WBRT plus EGFR-TKIs. Five studies were included in the comparison of WBRT plus EGFR-TKIs and WBRT alone. The combined HR for OS, intracranial PFS and extracranial PFS were 0.52 [95% CI 0.37–0.75; *P* = 0.0004], 0.36 [95% CI 0.24–0.53; *P* < 0.001] and 0.52 [95% CI 0.38–0.71; *P* < 0.001], respectively, which revealed a significant benefit of WBRT plus EGFR-TKIs compared with WBRT alone. The results indicated that EGFR-TKIs alone should be the first option for the treatment of NSCLC patients with multiple BM, especially with EGFR mutation, since it provides similar OS and extracranial PFS but superior intracranial PFS compared with WBRT plus EGFR-TKIs.

## INTRODUCTION

Brain metastasis in non-small cell lung cancer (NSCLC) is the most common intracranial metastatic tumor, and the incidence cases are more than half of all brain metastasis patients [1]. Approximately 25% to 40% of all NSCLC patients with brain metastases (BM) at some point in their disease course, and the risk is even higher in patients suffering from epidermal growth factor receptor (EGFR) mutation [2–4]. Furthermore, improved imaging in patients with prolonged survival due to the using of targeted systemic therapies in NSCLC with EGFR mutation is associated with a higher incidence of BM along with the disease process [5].

Therapeutic strategy for patients with BM vary according to the lesion number and size. Stereotactic radiosurgery (SRS) was always suitable for the patients with potentially superior prognosis [6]. However, whole brain radiation therapy (WBRT) has been regarded as the standard treatment for the tumor with a big size or those with > 3 lesions, but commonly leads to neurologic sequelae, such as cognitive impairment. Most chemotherapeutic drugs are effectiveness in treatment of NSCLC with BM except brain radiotherapy. Though blood brain barrier (BBB) around brain metastasis is damaged, the concentration of chemotherapeutic drugs in brain is still not high enough, which is possibly caused by the pump-out of chemotherapeutic drugs by tumor cells through efflux pump. As a small molecule compound, EGFR-tyrosine kinase inhibitors (EGFR-TKIs) can penetrate through the BBB in certain proportions, becoming an important theoretic mechanism for the treatment of NSCLC patients with BM. A previously published review demonstrated the intracranial efficacy of EGFR-TKIs which was globally superior to the efficacy of standard chemotherapy [7].

In recent years, researchers have utilized EGFR-TKIs to treat patients with brain metastasis [8–10]. Approaches adopted have included evaluating the role of EGFR-TKIs alone or with the concurrent use of EGFR-TKIs inhibition and WBRT in patients with brain metastases selected/unselected for EGFR mutation status. Several retrospective studies have compared the clinical outcomes of WBRT plus EGFR-TKIs and EGFR-TKIs alone in NSCLC patients with BM. Seonggyu et al. reported that no significant differences were found between the WBRT plus EGFR-TKIs and the EGFR-TKIs alone regarding intracranial PFS, extracranial PFS and OS [11]. However, in another study by Qinghua Xu et al., they found that compared with WBRT alone and EGFR-TKIs alone, patients who received concomitant WBRT and EGFR-TKIs had the longest median survival time [12]. The discrepancy can be ascribed to the limited sample size of those studies. Meta-analysis was therefore urgently needed to systematically assess the quality of available evidence and make a scientific conclusion about WBRT plus EGFR-TKIs compared with EGFR-TKIs alone and WBRT alone in treating BM from NSCLC.

The purpose of the present study included two meta-analysis was to compare overall survival (OS) and intracranial/extracranial progression-free survival (PFS) outcomes of NSCLC patients with BM who received WBRT plus EGFR-TKIs, WBRT alone or EGFR-TKIs alone. Through the two pooled analysis, we hoped to form a consensus about whether WBRT plus EGFR-TKIs, WBRT alone or EGFR-TKIs alone is superior for NSCLC patients with BM.

## RESULTS

### Characteristics of included trials

**Figure 1:**
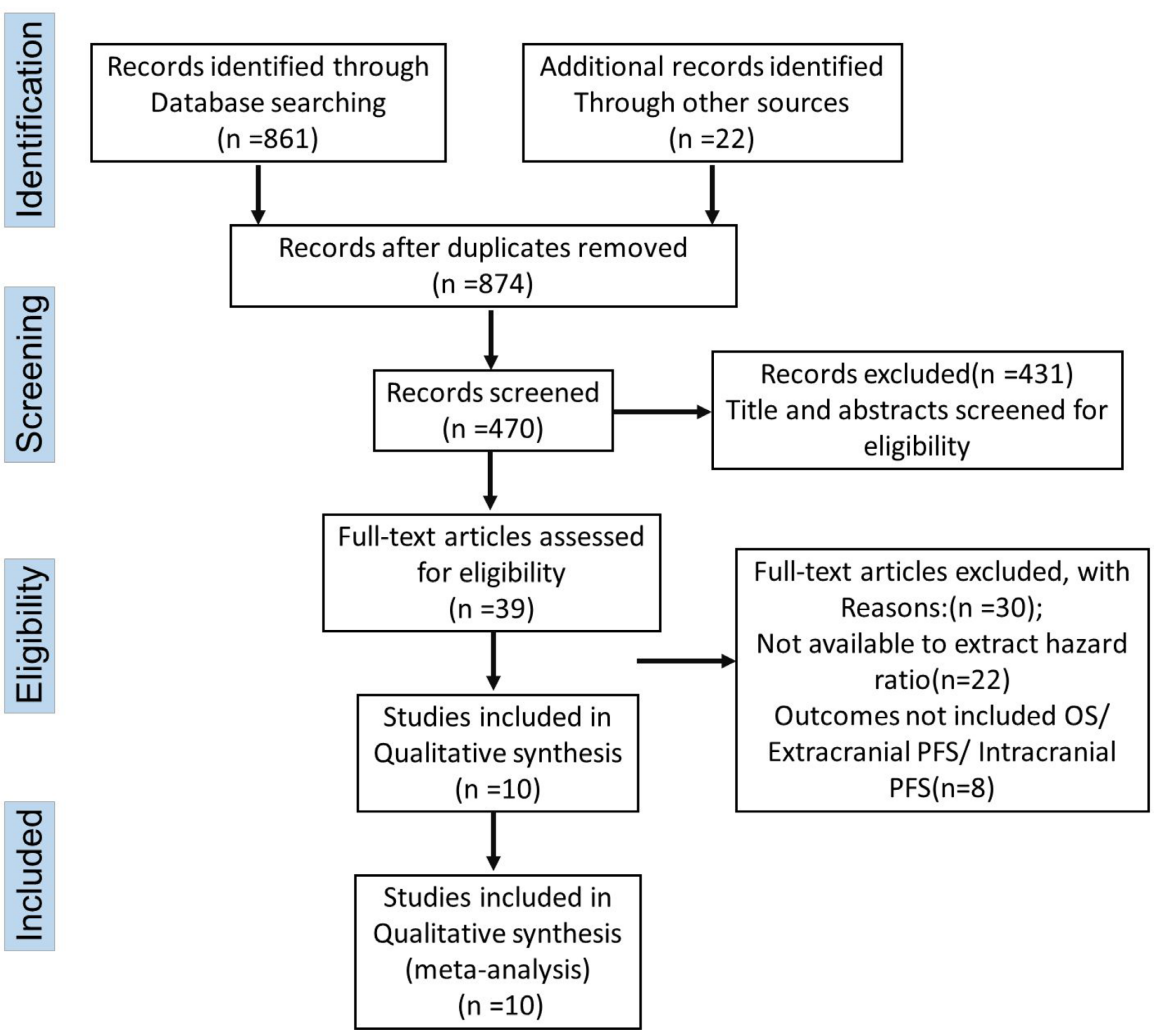
Flow chart of the literature retrieval according to the PRISMA statement

A total of 861 records were identified from electronic databases and 10 references were tracked. Finally, five studies involving 598 patients were included in the study to compare clinical outcomes between WBRT plus EGFR-TKIs and EGFR-TKIs alone [11, 13–16]. In the meta-analysis to compare clinical outcomes between WBRT plus EGFR-TKIs and WBRT alone, 5 studies with 443 patients were included [12, 17–20]. The search results and selection details are shown in Figure 1. Details for each study, including the, the status of EGFR mutation of primary lesion, the treatment procedure, the research year rang, the tumor stage for each trial and the publication year of the study were shown in Table 1.

**Table 1:** Description of studies comparing WBRT+TKIs between TKIs or WBRT only

Author	Year	WBRT+TKIs vs TKIs	WBRT+TKIs vs WBRT	Research year range	Follow up (m)	Tumor stage	Percentage patients with EGFR mutation in primary lesion	WBRT	TKIs	Research type	NOS score
Yin-Duo Zeng [15]	2012	45vs45		2005–2009	1–60	NA	12/90	40 Gy/20f/4w	gefitinib (250 mg) per day	retrospective	7
Yang-Si Li [14]	2016	33vs55		2011–2015	1–40	IV	100%	30 Gy/10 day fractions	gefitinib/erlotinib/icotinib/afatinib and sorafenib	retrospective	8
Tao Jiang [13]	2016	51vs116		2012–2015	1–40	IV(M1b)	100%	30 Gy/10 day fractions	gefitinib (250 mg) or erlotinib (150 mg) or icotinib (150 mg)per day; oral delivery	retrospective	7
Seonggyu-Byeon [11]	2016	57vs57		2005–2013	0.4–47.9	IIIB/IV	100%	2000 cGy for 5 days over a single week	gefitinib (250 mg) or erlotinib (150 mg) per day; oral delivery	retrospective	6
Yong-shun Chen [16]	2016	53vs79		2008–2014	3–80	IV	100%	30 Gy/10f for 5 days per week, up to 2 weeks	250 mg gefitinib or 150 mg erlotinib	retrospective	7
T Komatsu [19]	2013		19vs25	2005–2011	1–50	NA	5/44	30 Gy (range, 24-50 Gy)	gefitinib(250 mg) or erlotinib(150 mg)	retrospective	7
Y Cai [20]	2013		65vs92	2009–2012	1–26.6	NA	43/157	29.37∼41.24 Gy, 3 Gy/d, 5 times/week	gefitinib (250 mg) or erlotinib (150 mg)	retrospective	6
H Zhuang [17]	2013		23vs31	2009–2011	1–30	I-IV	11/54	30 Gy/10f, 5 days per week, up to 2 weeks	150 mg erlotinib	retrospective	6
SM Lee [18]	2014		40vs40	2009–2010	1–15	NA	1/35	20 Gy in 5 daily fractions	150 mg erlotinib	randomized	
Q Xu [12]	2015		42vs66	2006–2013	1–48	NA	11/108	30 Gy/10fx	gefitinib (250 mg) or erlotinib (150 mg)	retrospective	8

TKIs: epidermal growth factor receptor tyrosine kinase inhibitors; WBRT: whole brain radiotherapy; Gy: gray (absorbed dose); w: week; d: day; fx: fractions; NOS score: Newcastle-Ottawa Scale score; NA: not available.

### WBRT plus EGFR-TKIs versus EGFR-TKIs alone

**Figure 2:**
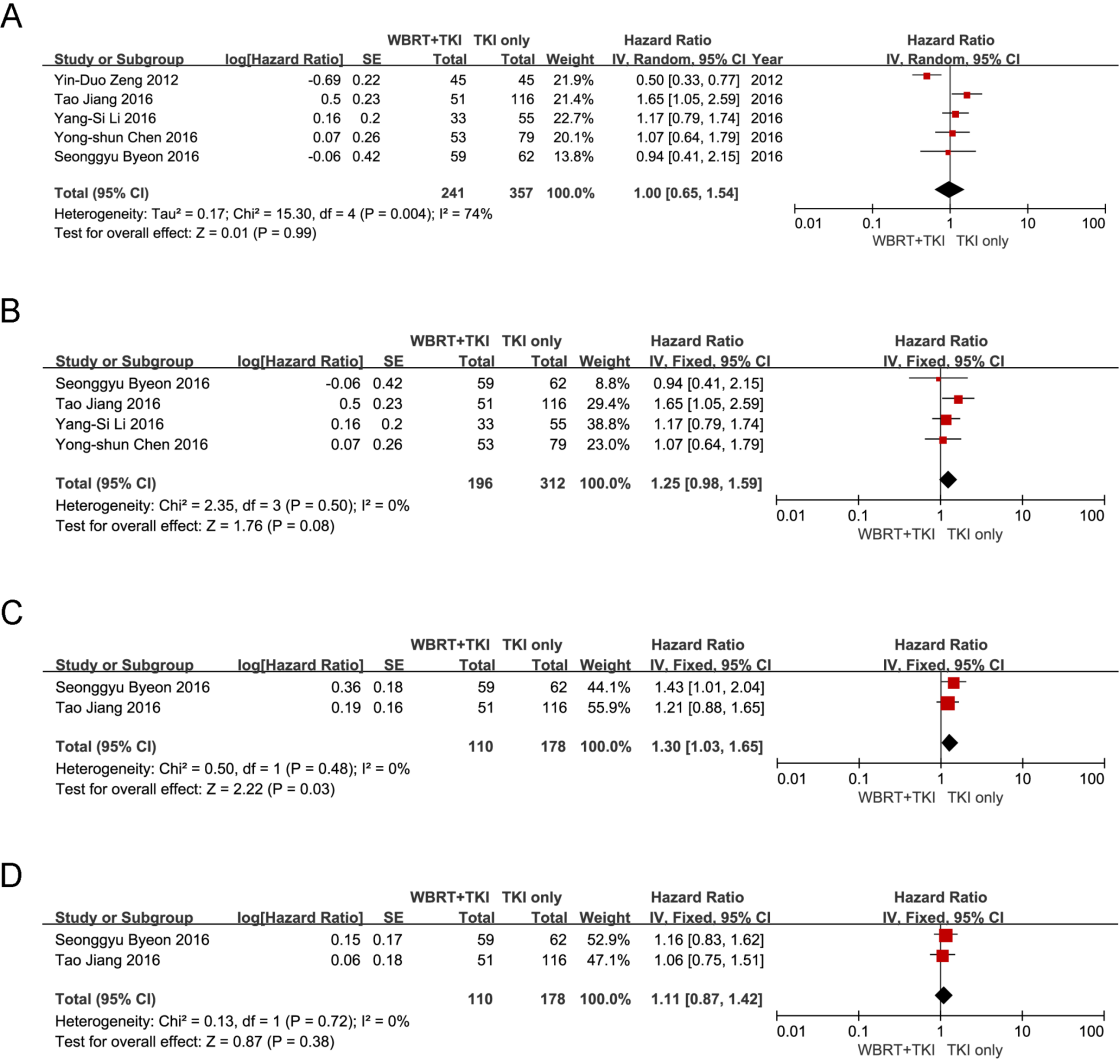
(**A**) Forest plot of comparison: the OS of WBRT+TKIs versus TKIs only in NSCLC patients with BM. Five studies were included. (**B**) Forest plot of comparison: the OS of WBRT+TKIs versus TKIs only in EGFR mutant NSCLC patients with BM. Four studies were included. (**C**) Forest plot of comparison: the intracranial PFS of WBRT+TKIs versus TKIs only in NSCLC patients with BM. Two studies were included. (**D**) Forest plot of comparison: the Extracranial PFS of WBRT+TKIs versus TKIs only in NSCLC patients with BM. Two studies were included.

After analyzed the practicable data in the existing researches, five studies [11, 13–16] involving 241 patients who received WBRT plus EGFR-TKIs were compared to 357 patients who received EGFR-TKIs alone to assess the overall survival from the date of treatment. As shown in Figure 2A, there was no significant difference in the OS (HR = 1.00 [95% CI 0.81–1.24; *P* = 0.99]). This result was coincident in the subgroup analysis which compared the OS between WBRT plus EGFR-TKIs and EGFR-TKIs alone in EGFR mutation NSCLC patients with BM (HR = 1.25 [95% CI 0.98–1.59; *P* = 0.08]) (Figure 2B). Intracranial PFS and extracranial PFS were reported in two studies involving 110 patients who underwent WBRT plus EGFR-TKIs and 178 patients who underwent EGFR-TKIs alone. As shown in Figure 2C, the meta-analysis revealed that compared with WBRT plus EGFR-TKIs for NSCLC patients with BM, the EGFR-TKIs alone treatment exhibited a superior intracranial PFS (HR = 1.30 [95% CI 1.03–1.65; *P* = 0.03]) (Figure 2C). However, there was no significant difference in extracranial PFS, as the combined HR was 1.11 [95% CI 0.87–1.42; *P* = 0.38] (Figure 2D).

### WBRT plus EGFR-TKIs versus WBRT alone

**Figure 3:**
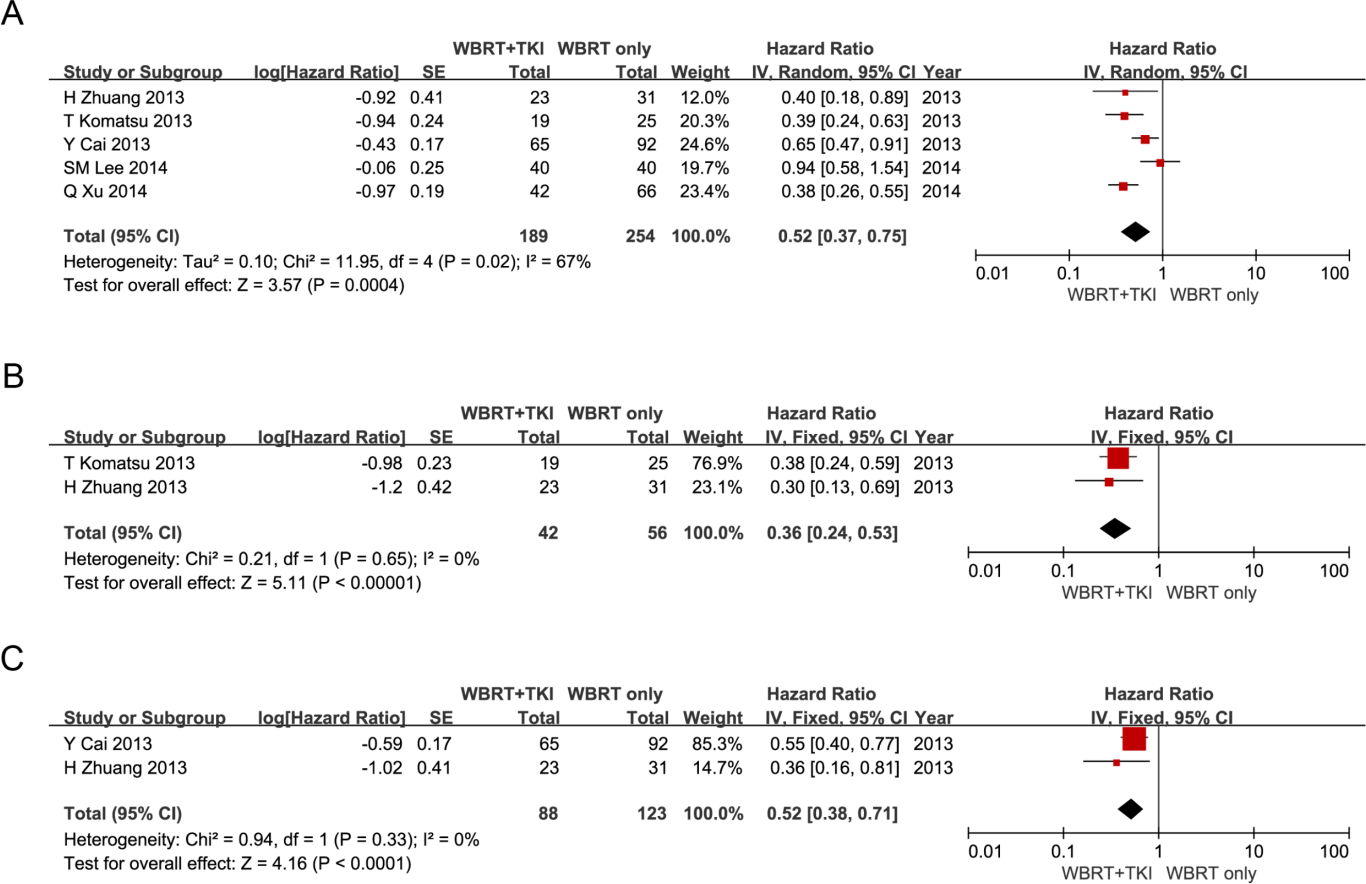
(**A**) Forest plot of comparison: the OS of WBRT+TKIs versus WBRT only in NSCLC patients with BM. Five studies were included. (**B**) Forest plot of comparison: the intracranial PFS of WBRT+TKIs versus WBRT only in NSCLC patients with BM. Two studies were included. (**C**) Forest plot of comparison: the Extracranial PFS of WBRT+TKIs versus WBRT only in NSCLC patients with BM. Two studies were included.

A total of five studies [12, 17–20] were included as data sources for the meta-analysis comparing clinical outcomes between WBRT plus EGFR-TKIs and WBRT alone. There were five studies involved in the analysis of the overall survival; 189 patients underwent WBRT plus EGFR-TKIs and 254 patients underwent WBRT alone. As shown in Figure 3A, the meta-analysis indicated that the OS for WBRT plus EGFR-TKIs was superior to WBRT alone in NSCLC patients with BM (HR = 0.52 [95% CI 0.37–0.75; *P* = 0.0004]). Two studies were included in the pooled analysis of intracranial PFS, with 42 patients treated by WBRT plus EGFR-TKIs and 56 patients treated by WBRT alone. The combined HR was 0.36 [95% CI 0.24–0.53; *P* < 0.00001] (Figure 3B). When comparing the extracranial PFS between WBRT plus EGFR-TKIs and WBRT alone, only two studies were included, with 88 patients treated by WBRT plus EGFR-TKIs and 123 patients treated by WBRT alone. The combined HR was 0.52 [95% CI 0.38–0.71; *P* < 0.0001] (Figure 3C). These pooled analysis revealed that compared with WBRT alone, WBRT plus EGFR-TKIs has a significant benefit for NSCLC patients with BM.

### Sensitivity analysis and publication bias

**Figure 4:**
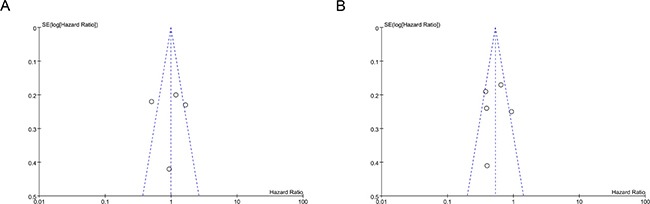
(**A**) Funnel plot of OS on the outcomes of the comparisons of WBRT+TKIs versus TKIs only in NSCLC patients with BM for the visual detection of systematic publication bias and small study effect. (**B**) Funnel plot of OS on the outcomes of the comparisons of WBRT+TKIs versus WBRT only in NSCLC patients with BM for the visual detection of systematic publication bias and small study effect.

To determine whether individual studies unduly influenced overall results, the analyses were repeated, excluding each study one at a time; no significant discrepancies in the outcomes were identified. The results were similar no matter random or fixed-effects models were performed. Publication bias was determined by asymmetry of the funnel plot which was used to estimate the precision of the trials (Figure 4A–4B). Each circle represents the treatment effect expressed as the logarithm of the HR of OS in each trial plotted against the standard error as a measure of study size. The perpendicular line exhibits the pooled estimate of the meta-analysis. Funnel plot analysis on the OS/PFS of comparisons did not indicate significant publication bias.

## DISCUSSION

The improvement of the clinical outcomes for NSCLC patients was owing to the deepen understanding the underlying molecular mechanism of lung cancer [17]. Recently, EGFR-TKIs have been considered as the standard first-line therapy for the EGFR mutant NSCLC patients, due to a series of large randomized phase III clinical trials uniformly revealed that treatment of EGFR-TKI achieved a higher response rate and longer PFS [18–20]. Although the low concentration ratios of brain-to-plasma for EGFR-TKIs (< 1–3%) have been reported, several prospective studies have shown that the treatment of EGFR-TKIs has a positive activity in NSCLC patients with multiple BM and EGFR mutation, with a response rate of up to 80% [21–23]. The status of the EGFR mutation in previous studies was always determined through detecting primary lesions; however, there is no evidence that whether NSCLC patients with BM should be treated with TKIs depends on the EGFR mutation status of primary lesions. The most direct reason is that we were unable to acquire metastatic lesions.

WBRT has been considered as the cornerstone treatment for multiple BM of NSCLC patients for decades [24, 25]. Currently, the efficacy of WBRT might be enhanced by EGFR TKI based on some reported evidence. Preclinical data reported by Zhuang H et al. and Chinnaiyan P et al. suggested that EGFR TKI synergizes radiation therapy in tumor control [26, 27]. According to some published clinical studies, compared with WBRT alone, EGFR TKI plus WBRT demonstrated an improved therapeutic effect in multiple BM of NSCLC patients [28, 29]. In addition, some studies demonstrated that in NSCLC patients with EGFR mutation and multiple BM, even EGFR-TKIs alone promised similar survival effects compared with WBRT plus EGFR-TKIs [11, 13]. However, the combination of WBRT and TKI remains questionable, especially considering that some studies reported that WBRT not only did not prolong the OS but also shortened the PFS. Therefore, we performed this meta-analysis to combine applicable studies to further examine the published data on this topic. This is the first meta-analysis on the combined OS/PFS of WBRT plus EGFR-TKIs versus WBRT alone and EGFR-TKIs alone for NSCLC patients with multiple BM.

In the meta-analysis comparing OS between WBRT plus EGFR-TKIs and EGFR-TKIs alone, no significant difference was found. To identify whether the results were influenced by the EGFR mutant status, we performed a subgroup analysis. Depending on the results of subgroup analysis in EGFR mutant NSCLC patients with multiple BM, we considered that EGFR-TKIs alone yielded a superior OS for WBRT plus EGFR-TKIs, but there were no significant statistic differences. Furthermore, compared with WBRT plus EGFR-TKIs, the EGFR-TKIs alone revealed a significant superiority for intracranial PFS. However, patients who underwent EGFR-TKIs alone did not exhibit any significant differences for extracranial PFS when compared to patients who underwent WBRT plus EGFR-TKIs. In addition, according to the meta-analysis comparing the OS/intracranial PFS/extracranial PFS between WBRT plus EGFR-TKIs and WBRT alone, we noticed that WBRT plus EGFR-TKIs is associated with significantly better OS, intracranial PFS and extracranial PFS in NSCLC patients with multiple BM.

William J.M et al found that upfront radiotherapy yield better outcomes for brain metastases in EGFR mutant NSCLC patients, the median OS in patients receiving upfront WBRT was 30 months, while the median OS was 25 months in those undergoing upfront EGFR-TKIs [30]. However, it is still unclear whether there are superior OS for upfront of WBRT followed by EGFR-TKI compared with WBRT combined with EGFR-TKIs according to the current studies. A prospective, multi-institutional randomized trial of upfront WBRT followed by EGFR-TKI versus WBRT combined with EGFR-TKI and TKI alone at iPFS and OS for NSCLC patients with multiple BM is urgently needed.

There are still several limitations in this study: (1) the level of evidence was relatively low, as most included articles were retrospective studies and whether clinical outcomes could be affected by undefined bias and/or confounding factors was unclear. (2) the radiation dose of WBRT and the type of EGFR-TKIs of the cohorts could not be collected and analyzed, which might have affected the survival of the NSCLC patients with multiple BM in some way. (3) we could not collect and analyse detailed the chemotherapy or surgery information of the cohorts, which might have influence on the outcomes of a few NSCLC patients in some respects. Despite the above limitations, the results of these two meta-analysis provided important evidence comparing the survival outcomes of WBRT plus EGFR-TKIs, EGFR-TKIs alone and WBRT alone in NSCLC patients with multiple BM.

In conclusion, these two meta-analysis revealed two important findings: (1) WBRT plus EGFR-TKIs produce significant superior OS, intracranial PFS and extracranial PFS to WBRT alone for NSCLC patients with multiple BM. (2) EGFR-TKIs alone produce similar OS and extracranial PFS but superior intracranial PFS with WBRT plus EGFR-TKIs in NSCLC patients with multiple brain metastases.

Therefore, for the therapy of NSCLC patients with multiple BM, especially those with EGFR mutation, the first choice should be EGFR-TKIs alone when it is available.

## MATERIALS AND METHODS

This study was designed according to the PRISMA statement [31]. To effectively identify included articles in our meta-analysis, a structured literature retrieval protocol was formulated according to the recommendations from the Cochrane Collaboration. All the objective, inclusion and exclusion criteria, primary and secondary outcomes, and the methods of synthesis were pre-specified before the analyses.

### Search strategy for published studies

We accomplished a literature retrieval for original researches by searching multiple electronic databases, including PubMed, MEDLINE, Embase, and ISI Web of Science, from their dates of inception to May 2017 using the following keywords related to lung cancer, WBRT, EGFR-TKIs, brain metastases and radiotherapy. The search and identification process was independently conducted by two authors (Hong Zheng and Quan Xing Liu). According to a standardized approach, and the selection of each study was reached by discussion. For purpose of acquiring the maximum sensitivity, we implemented the searching strategy as: “lung cancer” [all fields] AND “brain metastases” [all fields] AND “WBRT, radiotherapy” [MeSH Terms] OR “TKI” [all fields] OR “Gefitinib, Icotinib, Erlotinib” [all fields]. All the researches were filtered according to inclusion and exclusion criteria.

### Selection criteria

Researches accorded with the following inclusion criteria were considered eligible and included in this meta-analysis: (1) all the patients included had NSCLC with multiple BM (> 3); (2) study designs were prospective cohort studies or case-control studies; (3) provided data on OS or PFS; (4) intervention: WBRT plus EGFR-TKIs alone and control: EGFR-TKIs or WBRT alone. Researches were excluded depended on the reasons listed below: (1) reviews, commentaries, editorials, case reports, and letters; (2) lacked key information for the calculation of methods; (3) published duplicate studies with the accumulating numbers of patients or increased lengths of follow-up were deliberated, only the most informative article was included in this meta-analysis.

### Quality assessment

Quality of the included studies were assessed according to the Newcastle–Ottawa Scale (NOS) for the quality of cohort and case–control studies [32]. A star system for the NOS (range, 0–9 stars) was developed for the evaluation. The values for the included studies are shown in Table 1.

### Data extraction and critical appraisal

OS and PFS were the primary outcomes. All data for analyzing were extracted from included eligible articles (all available texts, tables and figures). Each retrieved article was reviewed by two independent investigators. A third expert adjudicator and discussion were performed if there were discrepancies between the two reviewers.

### Statistical analysis

The meta-analysis was performed by combining the results of the reported OS or PFS. The log (hazard ratio) [ln (HR)] and its standard error (SE) were used as the outcome measure for the combined data. Hazard ratio (HR) and associated variance data in each included study were obtained or calculated according to the techniques described by Tierney and Stewart [33]. Because the HR of OS/PFS could not obtained in some studies directly, data were extracted from the Kaplan–Meier survival curves of these studies to calculate the HR and SE of OS/DFS/CSS. Kaplan–Meier curves were read by Engauge Digitizer version 4.1 [34]. The calculations were performed independently by two researchers, and discrepancies were discussed to reach a consensus. The summary statistical analysis was conducted with Review Manager Version 5.1.2 (Cochrane Collaboration, Software Update, Oxford, United Kingdom). The heterogeneity between trials was assessed by using the Chi-square statistic; I^2^ less than 50% and a *P* value greater than 0.10 suggested that there was no statistical heterogeneity. The inverse variance fixed effects model was applied for meta-analysis. And the inverse variance random effects model was used when clinical characteristics and methodology did not show great differences, I^2^ was greater than 50% and the *P* value was less than 0.10. The influence of the study regarding overall effect size was identified by sensitivity analysis.
